# On the Wasting Diseases of Infants and Children

**Published:** 1871-06

**Authors:** 


*On the Wasting Diseases of Infants and Children.* By EUSTACE SMITH, M.D., London. Second American, from the Second Revised and Enlarged English Edition.

---

This work is written by one who seems to have well improved the opportunities enjoyed by him, as physician to two of the large London Dispensaries for sick children.

The introductory chapter gives us some very important information as to the peculiarities of the secondary diseases of badly nourished children ; and especially as to the insensibility of the nervous system, and the infrequency of reflex convulsions, so common in the other diseases of childhood. After speaking of the importance of diagnosing the secondary diseases under consideration, he briefly reviews the symptoms.

From him we learn that the enlarged abdomen, so common in the diseases of children is, in the great majority of cases, *the result of flatus in the bowels.*

In the general treatment of wasting diseases, the author very pointedly condemns the use of tonics, before the derangements of the stomach and bowels are corrected. Among the external applications he very properly attaches much importance to friction, bathing and inunction.

Friction with cod liver oil is recommended as a valuable means of introducing nourishment into the system. This treatment should receive more attention than physicians have given it.

Of course our writer insists that express and specific directions for diet in treating the wasting diseases of infancy are as important as the directions for the drugs. Yet, how often is this forgotten!

I can only give a very brief abstract of the remaining chapters of the book.

The first chapter treats of simple Atrophy from insufficient food, and is devoted, principally, to some excellent directions as to diet, weaning, etc.

The second chapter treats of that common and troublesome disorder of children—chronic diarrahœa. This is an excellent chapter, giving, with sufficient fullness, the causes, complications, anatomical characters, diagnosis, prognosis, prevention and treatment. The treatment is judicious and simple, as are most good things. In the use of antacils, astringents and opiates, certain rules are laid down which would seem to be a correct guide, and the observance of which would prevent the too common practice of mixing up our remedies, and using first one thing and then another, without anything to guide us, as to when to continue, when to stop, or when to change. I can only say further of the treatment, that a noticeable feature is the absence of the time-honored calomel. But this *is* a good remedy nevertheless. The raw-meat plan of treatment is highly recommended in some cases.

The third chapter has for its subject, Chronic Vomiting. The treatment recommended is plain, simple and doubtless effectual; consisting mainly, as it does, in attention to the diet. In this disorder, calomel in minute doses is highly recommended, by inference, though not directly.

The fourth chapter, on rickets, is full, for the size of the work. It is a subject in which the writer appears to feel an interest. But I must refer to his work for his views.

The fifth chapter embraces inherited syphilis. The subject of the sixth is Mucous diseases. Of the seventh, worms. Of the eighth, chronic tuberculosis. Of the ninth, pulmonary phthisics. Of the tenth, tuberculi, zatun of glands ; and the eleventh, and last chapter is devoted to the diet of children in health and disease. The most important chapter in the book.

But the whole work is a valuable addition to our medical libraries, and is to be commended for its brevity, for its simplicity, and for the definiteness of its instructions, evidently the result of experience and close observation.

As the work is small, the price cannot be large, and every physician should add it to his library. This is the kind of books we want now. Books that give us the whole thing in a nut-shell, the *concentrated extract of wisdom and experience.* Henry Lea, publisher, Philadelphia.

